# Survival under anthropogenic impact: the response of dragonflies (Odonata), beetles (Coleoptera) and caddisflies (Trichoptera) to environmental disturbances in a two-way industrial canal system (central Poland)

**DOI:** 10.7717/peerj.6215

**Published:** 2019-01-09

**Authors:** Edyta Buczyńska, Paweł Buczyński

**Affiliations:** 1 Department of Zoology, Animal Ecology and Wildlife Management, University of Life Sciences in Lublin, Lublin, Poland; 2 Department of Zoology, Maria Curie-Sklodowska University Lublin, Lublin, Poland

**Keywords:** Cooling water, Odonata, Coleoptera, Trichoptera, Dredging, Canals, Odonata, Environmental factors, Insect assemblages

## Abstract

Ecological metrics and assemblages of three orders of aquatic insects (Odonata, Coleoptera and Trichoptera—OCT) in an industrial canal system affected by dredging were studied. Five sites (a river as a control site and canals) along the Vistula River in Central Poland were sampled during six sampling periods (2011 and 2013). Canonical correspondence analyses (CCA) was used to assess the influence of environmental variables on the distribution of 54 insect species in the following system of habitats—a river feeding the canals, river-fed inlet canals and outlet canals with cooling waters. Additionally, before and after control impact (BACI) was used to test for the impact of canal dredging in 2011 on the insect response metrics. Non-metric multidimensional scaling analysis differentiated insect assemblages of the three habitats and similarity percentage (SIMPER) indicated the species most responsible for the faunistic dissimilarities. Temperature was found to be a key factor governing the presence of insects in the outlet canals with cooling water. CCAs revealed that electrolytic conductivity (EC) and salinity had the greatest influence on the OCT fauna in the river and the inlet canals, whilst it was the dissolved oxygen and the level of development of aquatic plants that proved most important in the outlet canals. Modified ANOVAs showed that dredging significantly affected the mean species richness and the dominance in the canals. The changes in OCT species composition were highly informative. The comparison between tolerance patterns of the OCT orders against the five parameters (temperature, EC, total dissolved solids (TDS), pH and current) revealed that caddisflies are the most sensitive group, followed by Coleoptera while Odonata proved the most resistant. Dragonflies have the greatest potential to serve as bioindicators of industrially heated waters. The OCT fauna responded specifically to different environmental factors and stressors, it is strongly recommended to track the responses on different levels, not only metrics, but above all, species.

## Introduction

Two approaches prevail in the studies on how environmental factors influence aquatic invertebrates at different levels (in natural or human-induced gradients, often relating to extreme ranges): one is based on ecological (faunistic) metrics (Pollard & Yuan, 2010; Suriano et al., 2011), the other on taxonomic levels—species or higher units (Bonada et al., 2004; Haidekker & Hering, 2008). Both have their merits and disadvantages: with the former, one can make quicker assessments of general trends, while the latter is more tedious and presupposes familiarity with one or many taxonomic groups (especially competence in identifying species and knowledge of their ecological preferences), but is the preferred option for describing changes at the local level. Ecological metrics by their very nature generate rough inferences, from which more detailed studies very often show deviations; taxonomic level, depending on the scale applied, generalizes (level of higher order taxa) or describes just a fraction of the interrelationships in an ecosystem (species level). As bioindicators, for instance, caddisflies are universally regarded as a sensitive order; along with mayflies and stoneflies, separately or jointly (EPT—Ephemeroptera, Plecoptera and Trichoptera) they are frequently analyzed in the context of water quality assessment (Pollard & Yuan, 2010). Dragonflies serve as good indicators of human disturbance in aquatic habitats or ecological integrity of lotic ecosystems (Samways & Steytler, 1996; Chovanec & Waringer, 2001). At the same time, species-based research, both in the field and in the laboratory, has shown that trichopterans—particularly the rheophilic family Hydropsychidae, a very common research model—are surprisingly resistant to certain forms of pollution (Bonada et al., 2004; Chang et al., 2014). An analogous situation is found among the odonate family Gomphidae, where the relevant differentiation is recorded not only at the specific but even at the generic level (Bernard, Buczyński & Tończyk, 2002; Mandaville, 2002). Hence, the most acceptable alternative appears to be a mixed approach, that is, one that indicates general trends but at the same time supplies more concrete data at the level of assemblages or species. This is especially apparent in the case of aquatic insects, where knowledge of the tolerance range of particular species to a given set of habitat parameters, including pollution, is difficult to acquire and far from complete: the gaps relate especially to taxa that are less common, difficult to trap or to identify (Rosenberg & Resh, 1993; Carlisle et al., 2007; Graf et al., 2008; Suriano et al., 2011). Moreover, it is quite often the case that laboratory data on the preferences of organisms for a given factor, such as temperature, do not correspond to conditions in the field, especially at the assemblage level (Haidekker & Hering, 2008). Much research has also focused on the reaction of organisms to lethal effects, for example, in biomonitoring, while completely ignoring sub-lethal effects, which in practice, may be more important, as they warn of pollution before the risk of damage to the ecosystem is too high (Kefford, 1998; Sala et al., 2016).

Most studies of habitat factors governing invertebrate assemblages of watercourses have concentrated on natural waters like streams and rivers, and these relationships are fairly well understood (Kefford, 1998; Haidekker & Hering, 2008; Suriano et al., 2011; Dallas & Ross-Gillespie, 2015). Anthropogenic watercourses, directly affected by an industrial plant, are very seldom used as a research model. This may be because conditions in such waters are usually extreme, indeed, lethal to most aquatic organisms. Open, generally accessible systems with sub-lethal factors are exceptional and consequently there are few systems that can be juxtaposed against natural habitats. The present study focused on the system of canals (together with their small feeder river) used for manufacturing purposes by the Zakłady Azotowe, Puławy, a regional chemical factory. These canals were dredged during this project and this additional aspect made them highly suitable for investigating the influences of diverse environmental factors and stressors on aquatic insects in combination with environmental gradients, the high values of which are human-induced, and also with the gradients associated with the structural (hydrodynamic) features of these watercourses.

The worldwide increase in industrialization has led to ever larger volumes of cooling waters, polluted by industrial effluents, getting into surface waters. The water utilized for cooling purposes by plants like Zakłady Azotowe comes under the heading of thermal pollution. The unnaturally elevated physical and chemical parameters of such water may disturb embryonic development, larval growth, metamorphosis, metabolism, breeding and simply eliminate many aquatic invertebrates (Haidekker & Hering, 2008). Temperature alone is one of the key factors affecting aquatic organisms both directly and indirectly, governing as it does the values of other important parameters like oxygen, electrolytic conductivity (EC), total dissolved solids (TDS) and salinity. EC, TDS and salinity are the-three of the interlinked factors that are most often determined in the context of water enrichment or contamination caused by mining activities or agricultural practices (Fernández-Aláez et al., 2002; Luek & Rasmussen, 2017). At present there is a tendency in research to multiply factors (stressors) describing different spatial levels (from waters through catchment areas to regions), which means that caution is required when interpreting results. Close links between some physical and chemical factors, for example, EC and dissolved oxygen (DO), may also cause difficulty in correctly interpreting the response of invertebrates, since one factor can mask the other (Kefford, 1998); in such a situation, it is useful to apply relevant multi-dimensional analyses.

Undoubtedly, dredging can also be included into human-induced factors that negatively affect biota of aquatic ecosystems. Industrial canals are regularly dredged. In natural running waters dredging is usually treated as an unmitigated disaster that removes all organisms, invertebrates included, from their environment together with substrate, sediments and vegetation (Aldridge, 2000). Conversely, dredging can have a positive impact, as was discovered in the case of the aquatic insects of a small, regulated, lowland river (Buczyński et al., 2016; Dąbkowski et al., 2016; Zawal et al., 2016): their populations regenerated rapidly, and the post-dredging fauna exhibited a greater species richness and higher proportion of rheobiontic and rheophilous species than before dredging. In such case one might construe dredging as a kind of ecological restoration, at least in the initial phase of the recovery of the microhabitats and insect assemblages. In industrial canals, the potential ecological restoration is obviously not so important; here, interest focused on how recolonization was proceeding and on the reactions of various groups of invertebrates in two different, artificial habitats (river water vs. cooling water). The study aimed to find out whether the reaction of insects in man-made habitat (canals), which was regularly dredged along its whole length, was similar to that previously observed on the river, or whether, because of its extent and intensity, the effects of dredging were entirely negative.

In this study three orders of aquatic insects were selected—Odonata, Coleoptera and Trichoptera (OCT)—which have long been used as environmental state indicators, especially in the context of water pollution and habitat disturbance (Sahlén & Ekestubbe, 2001; Houghtona, 2004; Balian et al., 2008; Suriano et al., 2011; Chang et al., 2014; Kalaninová, Bulánková & Šporka, 2014; Álvarez-Troncoso et al., 2015). These three orders respond differently to the factors and disturbances being investigated. Odonata and Coleoptera are regarded as more tolerant to water pollution than Trichoptera (Chang et al., 2014). Life histories of these groups are also different: whereas in dragonflies and caddisflies only the larval stages are totally reliant on water, in beetles both larvae and adults exhibit such a dependence. Moreover, their dispersal (recolonization) potentials differ: dragonflies are the strongest fliers, whereas among beetles and caddisflies there are both strong and weak fliers (Buczyński & Przewoźny, 2010; Landeiro et al., 2012; Curry & Baird, 2015). Taking into consideration all the aforementioned differences one can expect that these three insect orders depend on different environmental factors and their tolerance patterns are also different. Additionally, data considering the tolerance ranges of aquatic insects obtained in a laboratory should be treated with caution when compared to the field-gathered information (Carver et al., 2009). In our study we intended to check whether the reactions of particular insect groups in the field are similar or different and whether these groups form the suspected “sensitivity” formation from the most to the least tolerant: Coleoptera-Odonata-Trichoptera (Chang et al., 2014). Our data could be used in the selection of insect groups as potential bioindicators in the management strategies of such industry-influenced ecosystems.

The objectives of the present study were to investigate:

- the extent to which the entomofauna of a system of industrial canals fed by river water and/or lying close to the zone of riverine influences resembled that of a river;
- the key environmental factors (physical and chemical properties of the water as well as structural features of the canals) responsible for the species variations among insect assemblages of artificial watercourses—separately in inlet river-fed canals and in outlet canals with cooling water—and in the river itself (treated as a control site);
- the occurrence (tolerance) patterns of the three analyzed insect orders against selected water parameters: temperature, EC, TDS, pH and current;
- the response of aquatic insects at different levels of their organization (species, assemblages and ecological metrics) to the dredging of a system of canals 2 years after impact and how it differed among the various orders.

## Material and Methods

### Study area and sites

The study was carried out in the Central Mazovian Lowland (East European Plain, central Poland), in the mesoregion of the Middle Vistula Valley, in the center of an extensive area of old glacial plains lying in the catchment area of the Vistula River (Wisła). Originally, this area was covered by extensive primeval forests, the remnants of which have survived only on infertile sandy soils. Currently, this land is intensively farmed, with well-developed industrial infrastructure (Kondracki, 2000). The latter include the Zakłady Azotowe chemical plant in Puławy, which has a system of canals supplying it with water for production purposes and removing cooling water directly to the largest Polish river, the Vistula.

**Figure 1:**
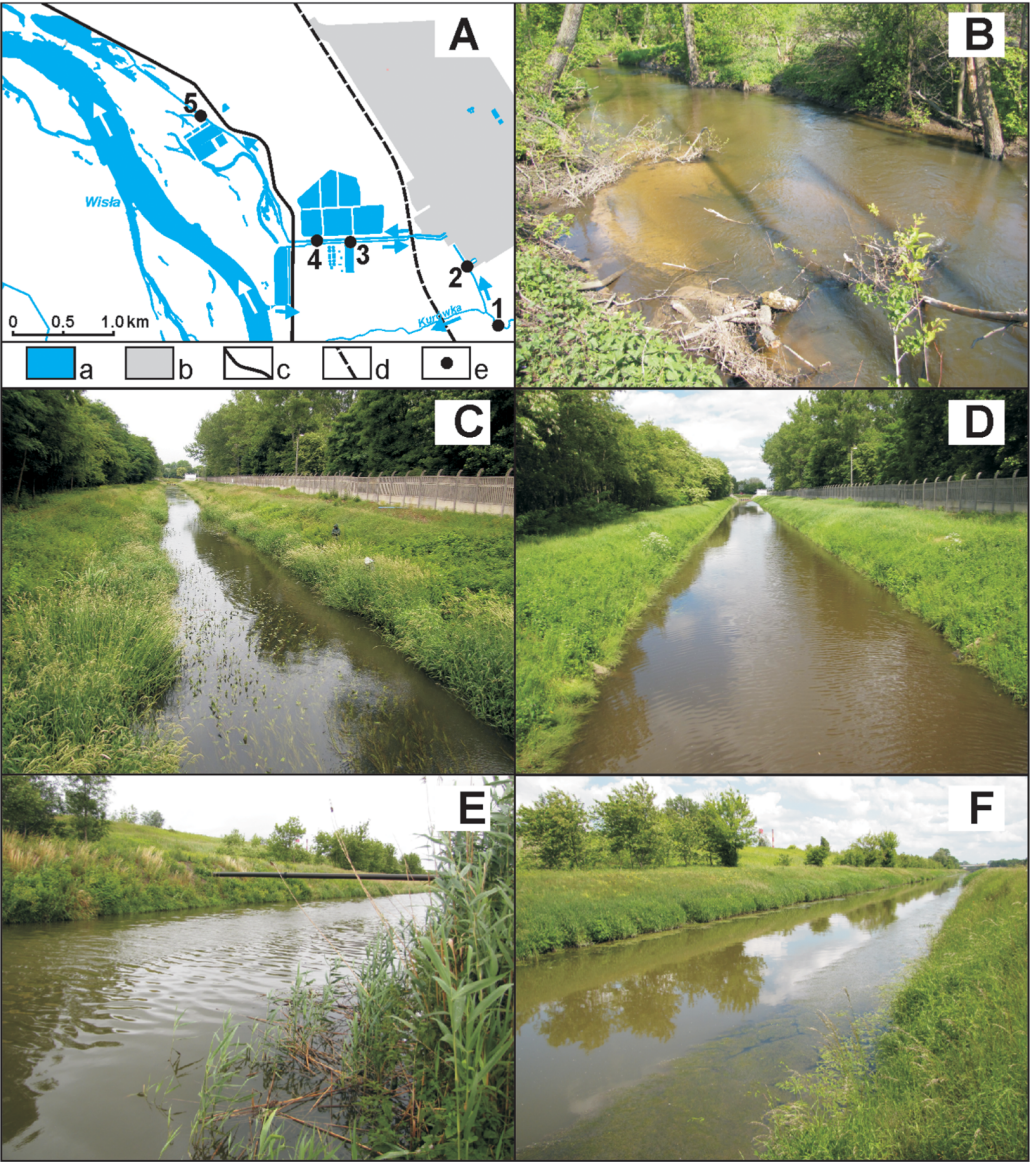
Study area and indicative sites. (A) General view: a, Surface waters; b, premises of the Zakłady Azotowe SA chemical plant; c, main road; d, main railway line; e, sites. (B) River—control site. (C) Inlet canal—site 2—before and (D) after dredging. (E) Outlet canal—site 4—before and (F) after dredging. (Photographs: Paweł Buczyński).

The study focused on five sites (Fig. 1, Appendix 1):

— site 1 (N 51°26′34.5″, E 21°58′41.1″)—the Kurówka River, feeding the outlet canal—a natural site (control)
— site 2 (N 51°26′53.0″, E 21°58′29.7″)—the inlet river-fed canal to the Zakłady Azotowe chemical factory in Puławy (ZA)
— site 3 (N 51°27′03.9″, E 21°57′04.4″)—the canal carrying water from the Vistula to the factory via a system of pumps and settling ponds
— site 4 (N 51°27′04.2″, E 21°57′07.1″)—an outlet canal (carrying cooling water and effluent from the factory)
— site 5 (N 51°27′45.1″, E 21°56′16.7″)—an outlet canal (carrying cooling water, industrial effluent and municipal sewage).

The sites are situated in the middle Vistula valley, on the floodplains of the Vistula and lower reaches of its tributary the Kurówka, on the terrain covered with a thick layer of sand. In this area, regulation of both rivers is minimal—both meander and periodically overflow their banks. Sites 3–5 lie on what was once riparian woodland (Kondracki, 2000), remnants of which have survived only near site 5; sites 3 and 4 now lie in an open, transformed industrial landscape. Site 1 (control) lies in a narrow river valley, surrounded by broad-leaved woodland growing on dunes bordering on the flood plain. Site 2 is similar in nature to sites 3 and 4. The Kurówka feeding the canal at site 2 is in good hydromorphological as well as physical and chemical condition (class II), but biologically poor (class IV); its overall state is regarded as bad (Żelazny, 2014).

### Collecting of aquatic insects samples

Samples were collected in 2011 (April, May, June) before impact and, following a 1-year break caused by the closure of the area by ZA, in 2013 (April, June, August) (Grupa Azoty Zakłady Azotowe “Puławy” S.A. field study approval number: ZK/6011/255/2018). Three transects covering the total area of ca. three m^2^ were established at every station on each sampling period. Three subsamples (90 in general) were collected using a hydrobiological sampler with a square frame and 250 μm mesh net for the representativeness of insect species. The samples were sorted in the laboratory and preserved in 70% ethanol. All specimens were identified to species level except for the *Anabolia* genus (representatives of the *A. laevis* and *A. furcata* species are not separable according to external morphology) and the larval stages of the *Agabus* and *Haliplus* genera.

### Environmental variables

The following environmental parameters were measured in situ using a HANNA HI 9828 multiparameter portable probe: water temperature (°C), pH, DO (ppm), EC (μS/cm), TDS (ppm) and salinity (PSU). The river flow rate (current) was measured using the float method. The coverage of aquatic and shore vegetation (emergent) was also estimated in situ and coded for subsequent statistical analysis as follows: 0—no aquatic plants/helophytes, 1—aquatic plants/helophytes: thin coverage, 2—aquatic plants/helophytes: moderate coverage, 3—aquatic plants/helophytes: dense coverage. The environmental features of each site are detailed in Table 1.

**Table 1:** Environmental variables (mean with standard deviations and abbreviations used in CCAs) for each sampling site before (2011) and after (2013) dredging.

Environmental variable	Abbreviation	Site and year (before/after dredging)									
		1. River (control)		2. Inlet canal		3. Inlet canal		4. Outlet canal		5. Outlet canal	
		2011	2013	2011	2013	2011	2013	2011	2013	2011	2013
Temperature (°C)	TEMP**	11.1 ± 4.2	13.8 ± 2.6	11.6 ± 3.6	13.8 ± 2.5	15.1 ± 4.7	18.0 ± 4.9	20.5 ± 3.5	25.0 ± 3.9	19.7 ± 3.7	24.3 ± 3.6
pH	pH**	8.22 ± 0.17	8.33 0.18	8.26 ± 0.10	8.22 ± 0.60	8.86 ± 0.46	8.10 ± 0.10	7.61 ± 0.19	7.65 ± 0.47	7.52 ± 0.54	7.68 ± 0.40
Dissolved oxygen (ppm)	O2	7.98 ± 3.04	7.63 ± 3.44	7.98 ± 3.1	6.99 ± 2.91	9.43 ± 2.2	7.66 ± 3.28	6.69 ± 1.7	5.79 ± 2.05	5.72 ± 1.5	4.77 ± 1.69
Electrolytic conductivity (μS/cm)	EC*	482 ± 18	565 ± 73	514 ± 34	579 ± 71	651 ± 64	658 ± 308	769 ± 67	707 ± 279	852 ± 64	769 ± 257
Total dissolved solids (ppm)	TDS*	240 ± 9	282 ± 37	256 ± 17	289 ± 35	338 ± 54	329 ± 154	384 ± 34	353 ± 140	426 ± 32	385 ± 129
Salinity (PSU)	SAL	0.32 ± 0.03	0.27 ± 0.04	0.36 ± 0.02	0.28 ± 0.04	0.40 ± 0.08	0.32 ± 0.16	0.41 ± 0.06	0.34 ± 0.15	0.47 ± 0.06	0.37 ± 0.12
Current (m/s)	CURR***	0.57 ± 0.13	0.64 ± 0.04	0.13 ± 0.09	0.17 ± 0.06	0.42 ± 0.11	0.52 ± 0.10	0.11 ± 0.06	0.16 ± 0.02	0.53 ± 0.09	0.52 ± 0.02
Aquatic plants	A_PLANTS	0.33 ± 0.58	1.00 ± 1.00	1.33 ± 2.31	1.00 ± 1.00	1.33 ± 0.58	0.00 ± 0.00	1.67 ± 0.58	2.00 ± 1.73	1.67 ± 0.58	1.33 ± 1.15
Riparian plants	R_PLANTS	0.00 ± 0.00	0.00 ± 0.00	0.33 ± 0.58	0.00 ± 0.00	0.00 ± 0.00	0.00 ± 0.00	0.33 ± 0.58	1.00 ± 1.00	0.00 ± 0.00	0.67 ± 0.58

**Notes:**

Significantly different variables among all sites are denoted by

* (*p* < 0.02).

** (*p* < 0.002).

*** (*p* < 0.0002).

### Data analysis

Dragonflies, beetles and caddisflies were analyzed at the level of species, orders, assemblages and faunistic metrics. Species richness (S), abundance (N), the Simpson dominance index (D), evenness according to Buzas and Gibson’s formula (E) and the Shannon–Wiener diversity index (H) were calculated on the basis of the OCT species matrix. Dominance at particular sites was also calculated based on the class ranges proposed by Biesiadka (1980) for aquatic insects: eudominants >10%, dominants—5.01–10%, subdominants—2.01–5% and recedents <2%.

To reveal the potential influence of the seasons at the control site, faunistic metrics and total abundance were *t*-tested. The same test was applied to find possible differences between the environmental variables in the canals before and after impact. Kruskal–Wallis tests indicated the environmental variables that varied significantly among all study sites. Then, each parameter (temperature, EC, TDS, pH and current) was used separately to detect and compare the response of each insect group: changes between total abundances of OCT were analyzed using Kruskal–Wallis tests, with Dunn’s tests being employed a posteriori. Box-whisker plots were used to compare and indicate the ranges and average values of selected parameters for particular insect groups.

Before and after control impact (BACI) is an analysis of variance technique which allows a potential influence of an environmental disturbance to be measured (Underwood, 1991). The BACI design included faunistic metrics (S, D, H, E) and the total abundance of insects (N). Two-way ANOVA took control/site and time effects into consideration: between the control site (river) and canal sites before and after impact, as well as between all artificial (canal) sites before and after impact. To meet the assumptions of the tests, data were ln transformed except in the case of the Shannon–Wiener diversity index (H). A Tukey-HSD test was performed on significant ANOVA results.

Non-metric multidimensional scaling (NMDS) indicated faunistic similarities between all sites in both study seasons. For this analysis, based on Jaccard’s index, pooled species data were used for three sampling periods before and after impact. In addition, hierarchical agglomerative clustering with the unweighted pair group method with arithmetic mean of pooling species was used to illustrate the similarity relationships between the five sites. The similarity percentage method (SIMPER) identified the species responsible for assemblage discrimination between riverine and canal sites (2, 3 and 4, 5 separately) as well as among canal sites before and after impact. The statistical analyses were performed using Statistica 13.1 and PAST 3.18 software (Hammer, Harper & Ryan, 2001).

To determine the significant factors responsible for the distribution of the insect assemblages (OCT and each order separately), multivariate ordination analyses were applied in three different habitats (natural—river, inlet river-fed canals, outlet canal with cooling water). Because no significant changes of environmental variables were found in the canals before and after impact (the only exception was the temperature at the outlet sites detected using U Mann–Whitney test: *p* < 0.0004), the focus was on the fauna of artificial habitats under the impact of systematic dredging, and not on single interventions. Analyses were preceded by detrended correspondence analyses, and if the gradient was longer than three SD, canonical correspondence analyses (CCA) were performed. To prevent neglect of the dredging impact, years were treated as dummy variables, as recommended by Lepš & Šmilauer (2003). The analyses involved nine environmental variables (six physical and chemical parameters of water and three structural features). Since EC and TDS values were highly correlated (*r* > 0.9), the second parameter was excluded from further analyses (Lepš & Šmilauer, 2003). Structural variables relating to plants were transformed into ordinal ones by coding and then treated as quantitative variables (Jongman, Ter Braak & Van Tongren, 1995). To test the significance of the environmental variables (*p* < 0.05), forward selection was used with the Monte Carlo permutation test. Multivariate statistics were carried out in CANOCO 4.5 (Ter Braak & Šmilauer, 2002).

## Results

**Figure 2:**
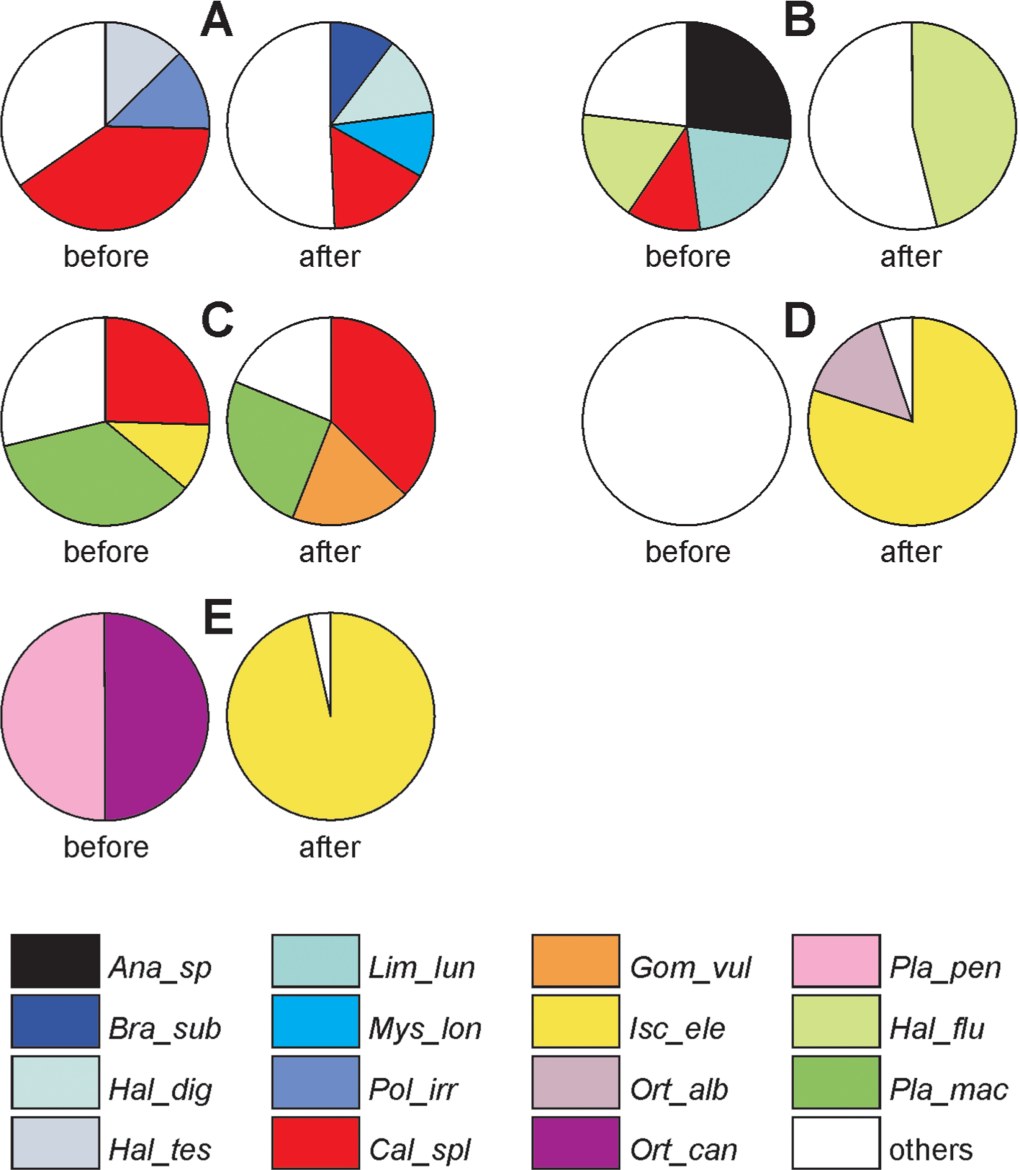
Percentage contribution of eudominant insect species at particular sites before and after dredging. (A) Site 1. (B). Site 2. (C) Site 3. (D) Site 4. (E) Site 5. Taxon codes are given in Table 2.

The total of 745 specimens from 54 taxa were found. Dragonflies were present in every habitat type studied: the river, the inlet river-fed canals and the outlet canals. The same applied to the beetles, except that there were very few of them (just two species) in outlet canals. Caddisflies occurred only in the river and the inlet canals (Table 2). In both years the same number of species was recorded in the river, but after dredging, the numbers of species in the canals rose—from 23 to 30 in the inlet canals and from two to seven in the outlet canals. *Calopteryx splendens* and four caddisfly species were the eudominants in the river (Fig. 2). At site 2, on an inlet canal, this class was represented by all three taxonomic groups; after dredging, almost half the fauna consisted of individuals of the *Haliplus fluviatilis* beetle. The inlet canal at site 3 was dominated by the dragonflies *Platycnemis pennipes*, *Calopteryx splendens* and *Ischnura elegans* (more than 70% of the fauna). After dredging, dragonflies were still dominant, but species composition had changed (*Calopteryx splendens* and *Gomphus vulgatissimus*), and the *Platambus maculatus* beetle had appeared. In the outlet canal, the nearest to chemical plant (site 4), no insects from the OCT assemblage were present at all; only after dredging did any appear—large numbers of *Ischnura elegans* and a few *Orthetrum albistylum.* Pre-dredging, the lower course of the outlet canal (site 5) was dominated by the *Orthetrum cancellatum* and *Platycnemis pennipes* dragonflies; post-dredging, large numbers of *Ischnura elegans* appeared, *Orthetrum cancellatum* was replaced by *Orthetrum albistylum* and numbers of *Platycnemis pennipes* fell dramatically.

**Table 2:** Trichoptera, Odonata and Coleoptera species collected at the five sites before (2011) and after (2013) dredging.

Taxon	Code	River		Inlet canals				Outlet canals			
		Site 1		Site 2		Site 3		Site 4		Site 5	
		2011	2013	2011	2013	2011	2013	2011	2013	2011	2013
Trichoptera											
*Anabolia furcata*/*A. laevis*	Ana_sp		2.3	**26.9**	3.30						
* Brachycentrus subnubilus*	Bra_sub		**10.3**								
* Glyphotaelius pellucidus*	Gly_pel			1.9							
* Halesus digitatus*	Hal_dig	3.6	**12.6**	1.9	1.10	6.4					
* H. tesselatus*	Hal_tes	**12.7**				5.3					
* Hydropsyche incognita*	Hyd_inc	1.8									
* H. pellucidula*	Hyd_pel		8.0	1.9							
* Lepidostoma basale*	Lep_bas	5.4	9.2	1.9	1.10						
* Limnephilus lunatus*	Lim_lun			**21.1**		7.4					
* Mystacides azurea*	Mys_azu				1.10						
* M. longicornis*	Mys_lon	1.8	**10.3**		1.10						
* Neureclipsis bimaculata*	Neu_bim			1.9		2.1					
* Oecetis furva*	Oec_fur					1.1					
* Polycentropus irroratus*	Pol_irr	**12.7**									
Odonata											
* Calopteryx splendens*	Cal_spl	**40.0**	**16.1**	**11.5**	4.40	**25.5**	**37.5**				
* C. virgo*	Cal_vir		9.2								
* Erythromma najas*	Ery_naj										0.1
* E. viridulum*	Ery_vir										1.5
* Gomphus vulgatissimus*	Gom_vul	5.4	6.9		3.30		**18.7**				
* Ischnura elegans*	Isc_ele		3.4		1.10	**10.6**			**80.0**		**96.6**
* Ophiogomphus cecilia*	Oph_cec		3.4	1.9			6.25				
* Orthetrum albistylum*	Ort_alb								**15.0**		0.3
* O. cancellatum*	Ort_can									**50.0**	
* Platycnemis pennipes*	Pla_pen	5.4			2.20	**35.1**				**50.0**	0.6
* Somatochlora metallica*	Som_met						6.2				
Coleoptera											
* Agabus* sp. (larva)	Aga_sp						6.2				
* Aulonogyrus concinnus*	Aul_con					1.1					
* Brychius elevatus*	Bry_ele		1.1								
* Deronectes latus*	Der_lat				1.10						
* Elmis maugetii*	Elm_mau	1.8									
* Enochrus melanocephalus*	Eno_mel								5.0		
* Graptodytes granularis*	Gra_gra			1.9		1.1					
* Haliplus confinis*	Hal_con				1.1						
* H. flavicollis*	Hal_fla			1.9	7.7						
* H. fluviatilis*	Hal_flu			**17.3**	**46.1**	1.1					
* H. variegatus*	Hal_var				3.3						
* Haliplus* sp. (larva)					2.2						
* Hydraena palustris*	Hyd_pal					1.1					
* Hydroporus glabriusculus*	Hyd_gla				1.1						
* H. incognitus*	Hyd_inc				2.2						
* Hygrotus impressopunctatus*	Hyg_imp	1.8									
* H. inaequalis*	Hyg_ina			1.9	1.1						
* H. versicolor*	Hyg_ver			1.9	1.1						
* Hyphydrus ovatus*	Hyp_ova	1.8			6.6						
* Laccobius minutus*	Lac_min		1.1		1.1						0.3
* Laccophilus hyalinus*	Lac_hya		5.75		2.2						
* L. poecilus*	Lac_poe				1.1						
* Limnebius parvulus*	Lim_par				1.1						
* Nebrioporus depressus*	Neb_dep			1.9							
* Noterus crassicornis*	Not_cra			1.9	1.1	1.1					
* Orectochilus villosus*	Ore_vil	1.8									
* Oulimnius tuberculatus*	Oul_tub	3.6									
* Platambus maculatus*	Plat_ma						**25.0**				
* Rhantus notaticollis*	Rha_not					1.1					

**Note:**

Eudominant species are shown in bold.

**Figure 3:**
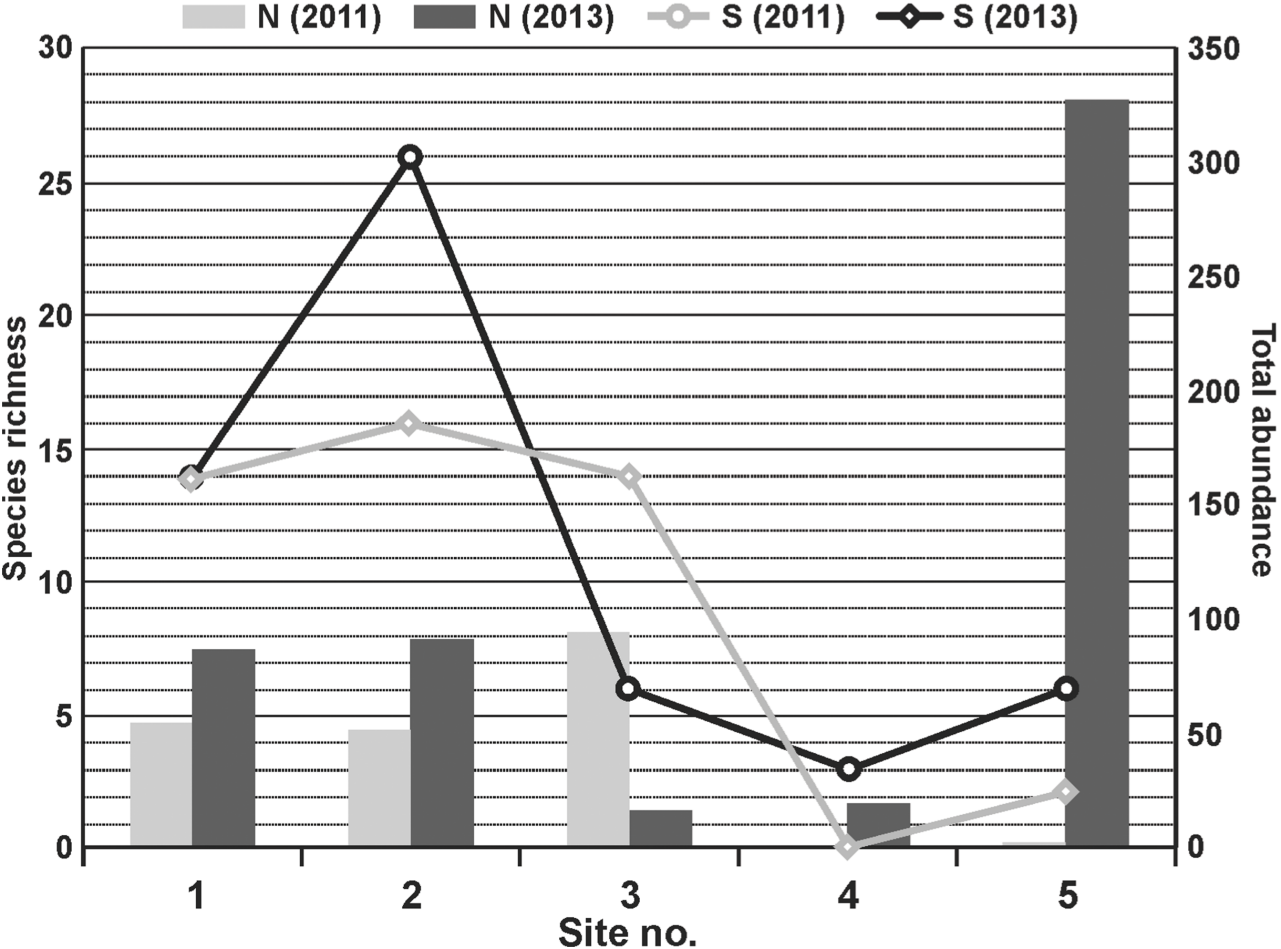
Changes in total abundance (N) and species richness (S) of aquatic insects at the five sites before (2011) and after (2013) dredging.

Before impact, the largest numbers of individuals and species were caught in the river and the inlet canals, both metrics indicated a reduction in richness and abundance in outlet canal habitats. After impact, the numbers of individuals were roughly the same as before impact in the river and the first inlet canal, they fell conspicuously in the second inlet canal, but reached record numbers (dragonflies were responsible for this rise) at site 5. The distribution of species richness after dredging was very close to that existing before impact, except that in 2013 it was more evenly balanced in the river-inlet canal system (Fig. 3). Values of the diversity index H (>2) were the highest for the control site and the inlet canal (site 2) before and after impact. The H value at site 3 before impact was only slightly lower (1.9) and dropped to 1.55 two years later. Generally lower values of H were recorded for the outlet canals: at site 4, H increased after impact from 0 to 0.6 whereas at site 5 it decreased from 0.6 to 0.19. Dominance declined by half in 2011 comparing to 2013 (*D* = 0.20 and *D* = 0.09, respectively). An increase was recorded in the inlet canals after impact (site 2 before and after: *D* = 0.16 and *D* = 0.23; site 3: *D* = 0.21 and *D* = 0.25). In both outlet canals, there was a sudden increase in this index, for which the very large numbers of *Ischnura elegans* were responsible, both before and after dredging, especially at site 5 (site 4: *D* = 0. and *D* = 0.66; site 5: *D* = 0.5 and *D* = 0.93).

**Figure 4:**
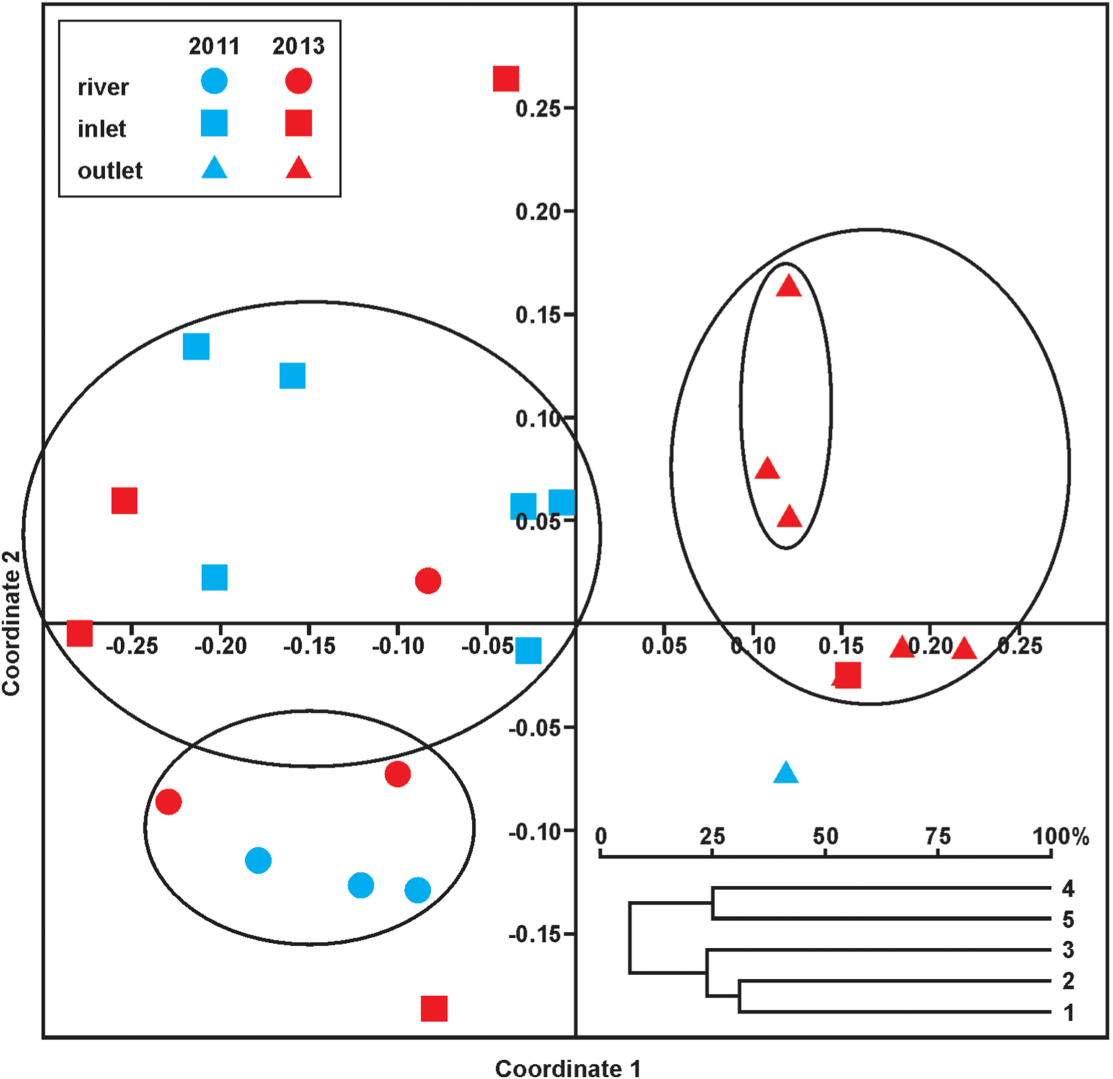
NMDS plot showing the faunistic similarities between the fauna of the control site (river), inlet and outlet canals before (2011) and after (2013) dredging. Stress value: 0.156. Calculations based on pooled data for the sampling periods (Jaccard’s index). Below: cladogram showing general faunistic similarities between all sites (pooled data from two years).

The NMDS plot (Fig. 4) illustrated the following relationships: the fauna of the river before and after impact was quite similar and closest to that of the inlet canals (left-hand side of the plot). With few exceptions, axis 1 separated the fauna of the river and river-fed inlet canals before and after dredging from the outlet canal fauna after impact. Axis 2, in turn, distinguished river sites (the lower left quarter) from inlet canals (mainly before impact) and the outlet canal sites after impact (the upper right quarter). The same index applied to the data from each site in both seasons showed that the faunistic similarity was the greatest between the river and inlet canal site 2 (31%), then between the two inlet canals (27%) and finally between the two outlet canal sites (25%). At the same time, the cladogram shows clearly that the sites were separated into two groups: the river together with the two inlet canals, and the two outlet canals.

Similarity percentage analysis (Table 3) showed that before dredging the quantitative faunistic similarity was the greatest between the river and the inlet canal sites, but negligible between the river and outlet canal sites. In the second season, after the intervention, the fauna of the inlet canals was less riverine in nature, but the similarity between the outlet canal sites was almost the same as before, even though a new species distinguishing these sites—*Ischnura elegans*—had made its appearance. After dredging, the faunistic similarity between the inlet and outlet canals increased significantly compared to before impact: the percentage of species responsible for most of the dissimilarity was now completely different. Before impact, *Calopteryx splendens* and *Limnephilus lunatus*, with the cumulative contribution of 40%, were responsible for this to the highest degree, whereas after impact these species were *Ischnura elegans* and *Haliplus fluviatilis* (cumulative contribution 51%). The differences between the two study seasons in the river itself were due solely to *Calopteryx splendens*. The same dragonfly species, together with *Haliplus fluviatilis*, contributed the most to the difference in the insect assemblage structure before and after impact in the inlet canals. *Ischnura elegans* and *Orthetrum albistylum* were the two dragonfly species most responsible for assemblage discrimination between the outlet canal sites before and after impact. At the same time, it should be noted that despite dredging, the latter sites retained the most similar fauna throughout the study area, an indication that hydrological continuity is of less importance than the kind of habitat.

**Table 3:** Results of a similarity percentage (SIMPER) analysis between the insect assemblages from three habitat types before (2011) and after (2013) dredging as well as within seasons.

Sites (habitat types)	Oad %	Species responsible the most for dissimilarity
Control × canals B/A		
River × Inlet	94	Cal_sple, Hal_flu
River 2011 × Inlet_2011	91	Cal_spl, Lim_par, Pla_pen
River 2013 × Inlet_2013	96	Cal_sple, Hal_flu
River × Outlet	99	Cal_sple, Isc_ele, Lep_bas
River 2011 × Outlet_2011	99	Cal_spl, Lep_bas
River 2013 × Outlet_2013	98	Isc_ele, Cal_spl
Among canals (B/A)		
Inlet × Outlet	80	Isc_ele, Hal_flu
Inlet 2011 × Outlet 2011	92	Lim_lun, Cal_spl
Inlet 2013 × Outlet 2013	79	Isc_ele, Hal_flu
Within habitat type (seasons and B/A)		
River 2011 × River 2013	87	Cal_spl
Inlet 2011 × Inlet 2013	91	Hal_flu, Cal_spl
Outlet 2011 × Outlet 2013	59	Isc_ele, Ort alb

Note:

Oad%—the average % of dissimilarity. The codes of species contributing the most to dissimilarity are given in Table 2.

No significant differences in the total abundance of OCT and faunistic metrics were found at the riverine control site between the two study periods, and the total species richness in 2013 was the same as in 2011 (Table 2). This confirmed that potential faunistic differences between both study periods (2001 and 2013) did not affect the results of subsequent comparative analyses. No statistically significant difference in the faunistic indices were recorded between the river and the inlet canals either (Table 4): they were consistent over time and space (*p* > 0.05), which endorses the assumption that the river would have a major influence on the fauna of the inlet canals. Significant differences did appear, however, when the fauna of the river was compared with that of the outlet canals, and also between the fauna of the inlet and outlet canals. Time and site significantly influenced the mean values of species richness and dominance index of the river and outlets; however, the BACI interactions were not significant. Moreover, the mean diversity index, which decreased after impact, was affected by the site factor. Two metrics changed significantly when the faunas of the canals before and after impact were compared: the mean number of species (pairwise comparisons based on Tukey’s HSD test: after inlet and after outlet—*Q* = 6.213, *p* = 0.0003) and the dominance index (pairwise comparisons based on Tukey’s HSD test: before outlet and after outlet—*Q* = 3.59, *p* = 0.042; after inlet and after outlet—*Q* = 6.52, *p* = 0.00021). The mean species richness in the inlet canals was significantly higher after impact in contrast to the sites situated on the outlet canal. The mean dominance index after impact increased at the inlet sites and decreased at the outlet canal sites. The single effect of site on the mean Shannon–Wiener diversity index was detected: it increased after impact in the inlets but decreased in the outlets. There were no significant differences (*p* > 0.05) between the mean evenness index and abundance.

**Table 4:** Results of two-way ANOVA tests on faunistic metrics and total abundances of OCT (Odonata, Coleoptera, Trichoptera) assemblages before and after dredging.

	R vs. canals						Among canals		
	R(C) × Inlet			R(C) × Outlet			Inlet × Outlet		
Metric	B/A	CI	Interaction (B/AxSite)	B/A	CI	Interaction (B/AxSite)	B/A	Site	Interaction (B/AxSite)
S	ns	ns	ns	0.042	0.003	ns	ns	0.0001	0.038
N	ns	ns	ns	ns	ns	ns	ns	ns	ns
H	ns	ns	ns	ns	0.0157	ns	ns	0.001	ns
D	ns	ns	ns	0.003	0.001	ns	ns	0.0001	0.006
E	ns	ns	ns	ns	ns	ns	ns	ns	ns

**Figure 5:**
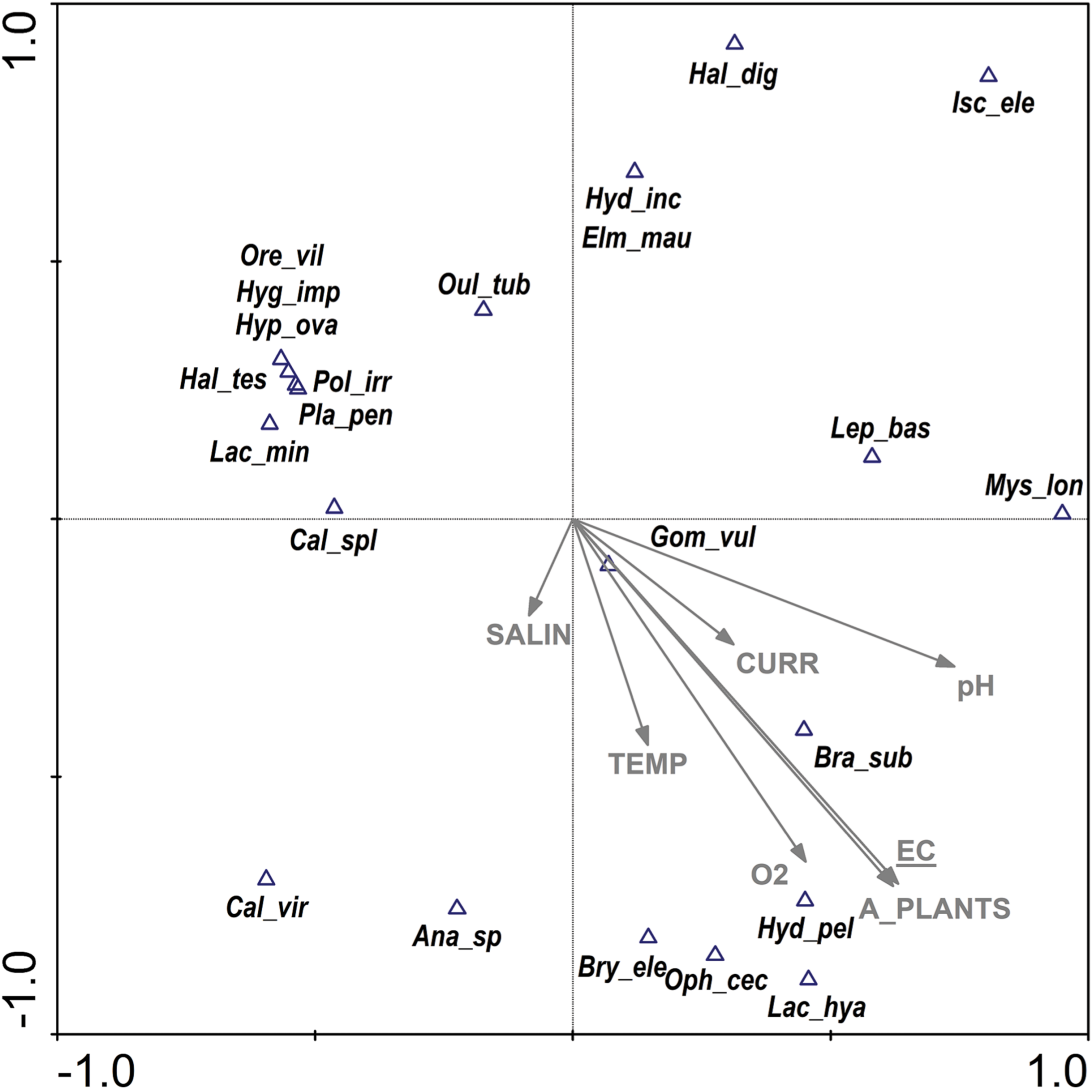
CCA ordination biplot showing the distribution of Odonata, Coleoptera and Trichoptera taxa of the river (control site) vs. environmental (physical, chemical and structural) variables. Eigenvalues: axis 1—0.70, axis 2—0.61, the total inertia—5.19. The statistically significant parameter (EC λa = 0.65, *p* = 0.002, *F* = 1.85) is underlined. The abbreviations for the variables and taxon codes are explained in Tables 1 and 2.

Under natural conditions (in the river), EC was the most important factor shaping the OCT assemblage (Fig. 5); the explanatory variables used in the analysis accounted for 57% of its variability. The CCA biplot showed that among all species two trichopteran species: *Brachycentrus subnubilus* and *Hydropsyche pellucidula* were positively correlated with this factor; the former with mean values of this parameter while the latter with the highest. The results for the three insect orders separately (Table 5) showed that the same parameters (EC, salinity) were responsible for the distribution of dragonfly and caddisfly species in the river. For beetles EC was the only key factor.

**Table 5:** Environmental variables (EV) significantly influencing the particular orders of insects (conditional effects) in the river, inlet and outlet canals, according to the CCA models.

Insect order	TVE (%)	EV	λa	*p*	*F*
River (control site)					
Odonata	87%	SAL	0.55	0.020	8.24
		EC	0.41	0.030	2.41
Coleoptera	78%	EC	1	0.014	1.37
Trichoptera	76%	EC	0.74	0.002	2.95
		SAL	0.52	0.044	2.34
Inlet canals (site 2 and 3)					
Odonata	72%	O_2_	0.21	0.048	2.44
Coleoptera	52%			ns	
Trichoptera	74%			ns	
Outlet canals (site 4 and 5)					
Odonata	92%	CURR	0.66	0.014	4.5
		O_2_	0.39	0.004	5.9
Coleoptera	–	–	–	–	–
Trichoptera	–	–	–	–	–

**Note:**

TVE (%), total variance explained; λa, increase in eigenvalue (additional fit); *p*, significance level of the effect tested by Monte Carlo permutation test; *F*, value of the F-ratio statistic. Abbreviations of environmental variables (EV) are given in Table 1.

**Figure 6:**
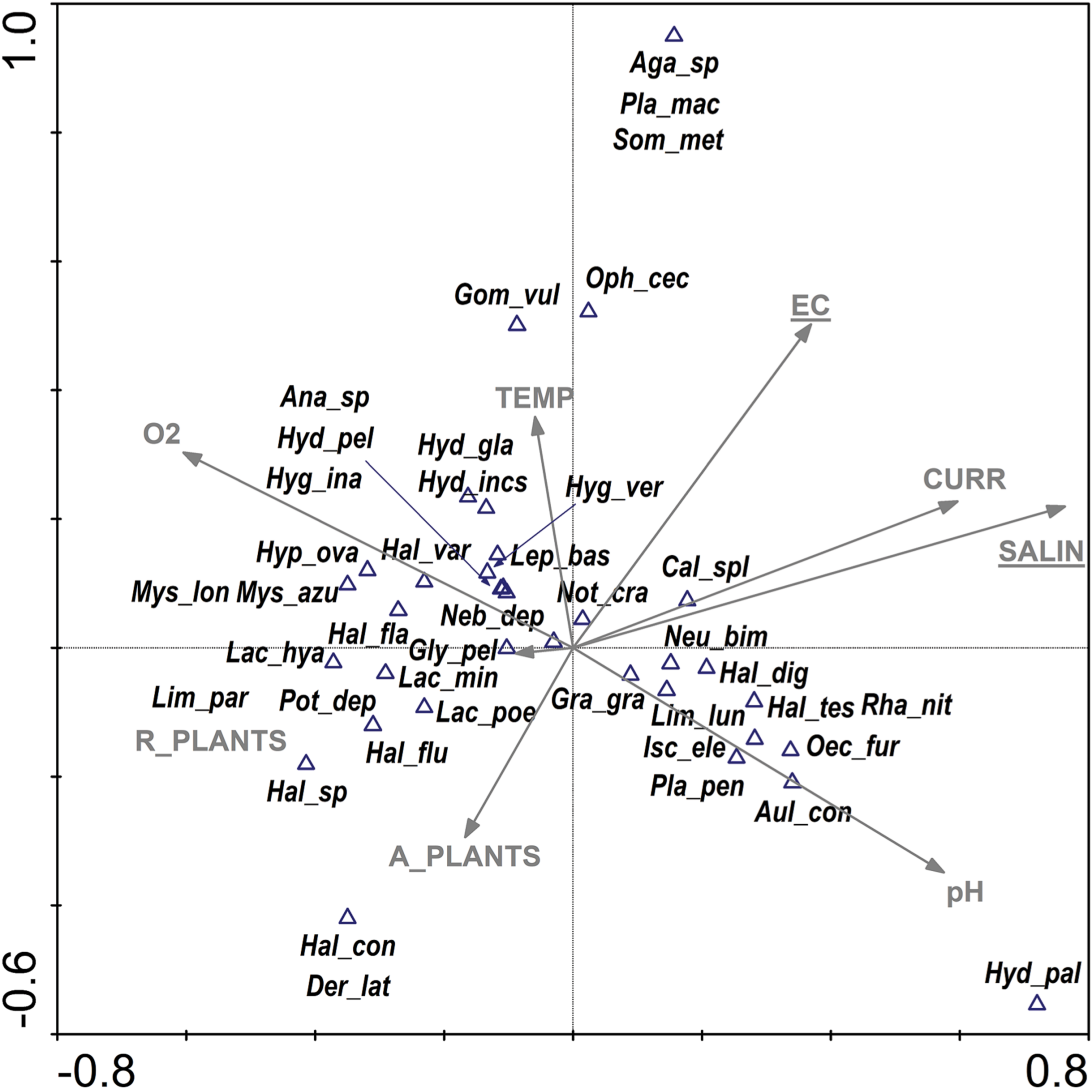
CCA ordination biplot showing the distribution of Odonata, Coleoptera and Trichoptera taxa of the inlet canals vs. environmental (physical, chemical and structural) variables. Eigenvalues: axis 1—0.73, axis 2—0.53, the total inertia—6.19. The statistically significant parameters (SALIN λa = 0.56, *p* = 0.010, *F* = 1.88; EC λa = 0.48, *p* = 0.046, *F* = 1.70) are underlined. The abbreviations for the variables and taxon codes are explained in Tables 1 and 2.

Salinity and EC were the most important parameters in the inlet canals and all the parameters used in the analysis explained 47% of the total variability among the species found (Fig. 6). *Calopteryx splendens*, *Neureclipsis bimaculata*, *Halesus digitatus* and *Graptodytes granularis* were most closely associated with lower values of salinity. *Noterus crassicornis* was the only species that was strongly positively correlated with lower EC. Much more species showed negative correlations with both parameters. Only one parameter—oxygen content (Table 5) was of significance to dragonflies in the inlet canals. No factor was found to be statistically significant in case of caddisflies and beetles.

**Figure 7:**
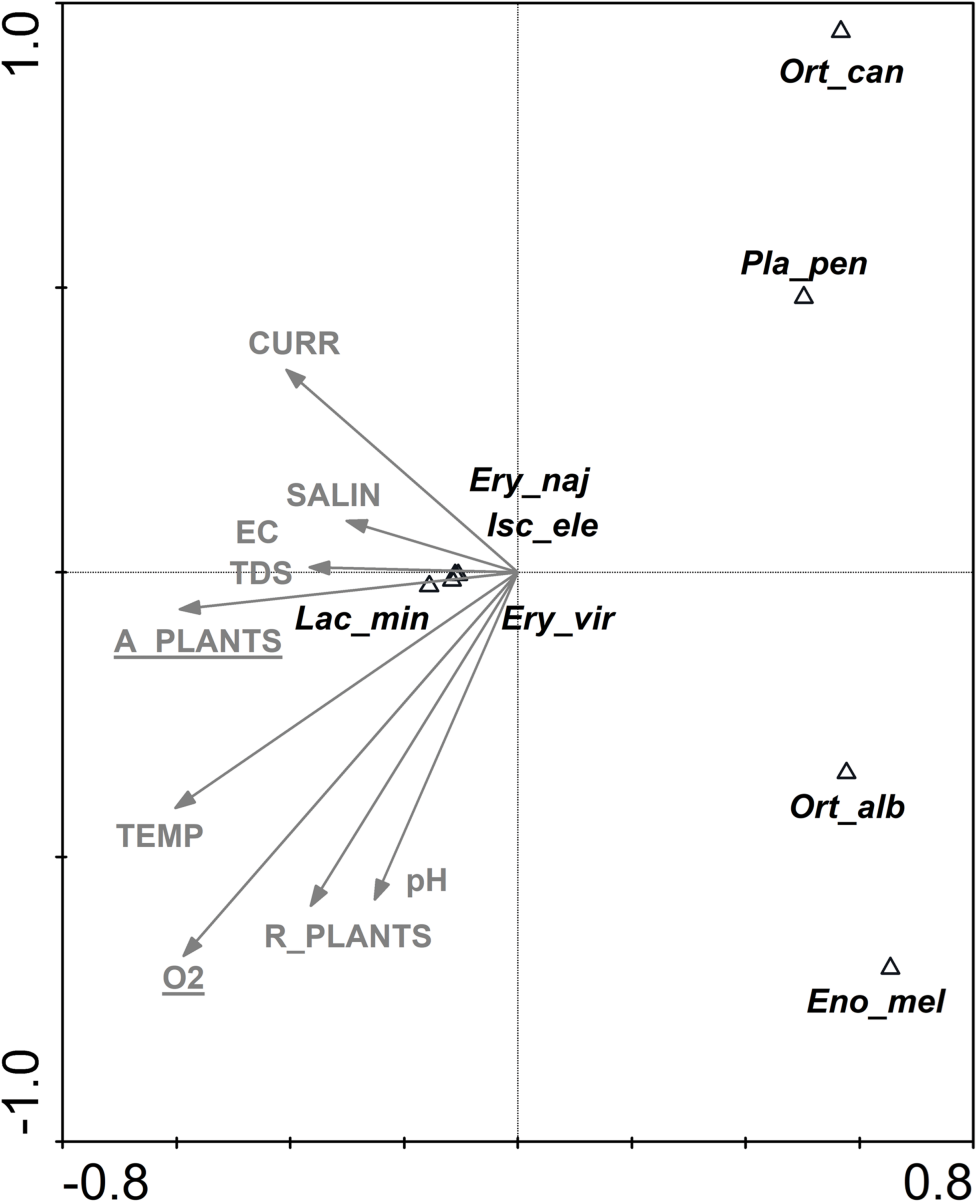
CCA ordination biplot showing the distribution of Odonata, Coleoptera and Trichoptera taxa of the outlet canals vs. environmental (physical, chemical and structural) variables. Eigenvalues: axis 1—0.92, axis 2—0.74, the total inertia—2.62. Statistically significant parameters (A_PLANTS λa = 0.61, *p* = 0.006, *F* = 5.37; O_2_ λa = 0.68, *p* = 0.014, *F* = 3.18) are underlined. The abbreviations for the variables and taxon codes are explained in Tables 1 and 2.

Two factors were responsible for the depauperate fauna in the outlet canals: aquatic vegetation and oxygen (Fig. 7)—they explained 93% of the total species variability. Three dragonfly species (*Erythromma viridulum*, *E. najas* and *Ischnura elegans*) and one species of beetle (*Laccobius minutus*) were the most closely associated with the coverage of aquatic vegetation. A separate CCA analysis for dragonflies indicated oxygen and current speed to be the most significant for this group (Table 5).

**Figure 8:**
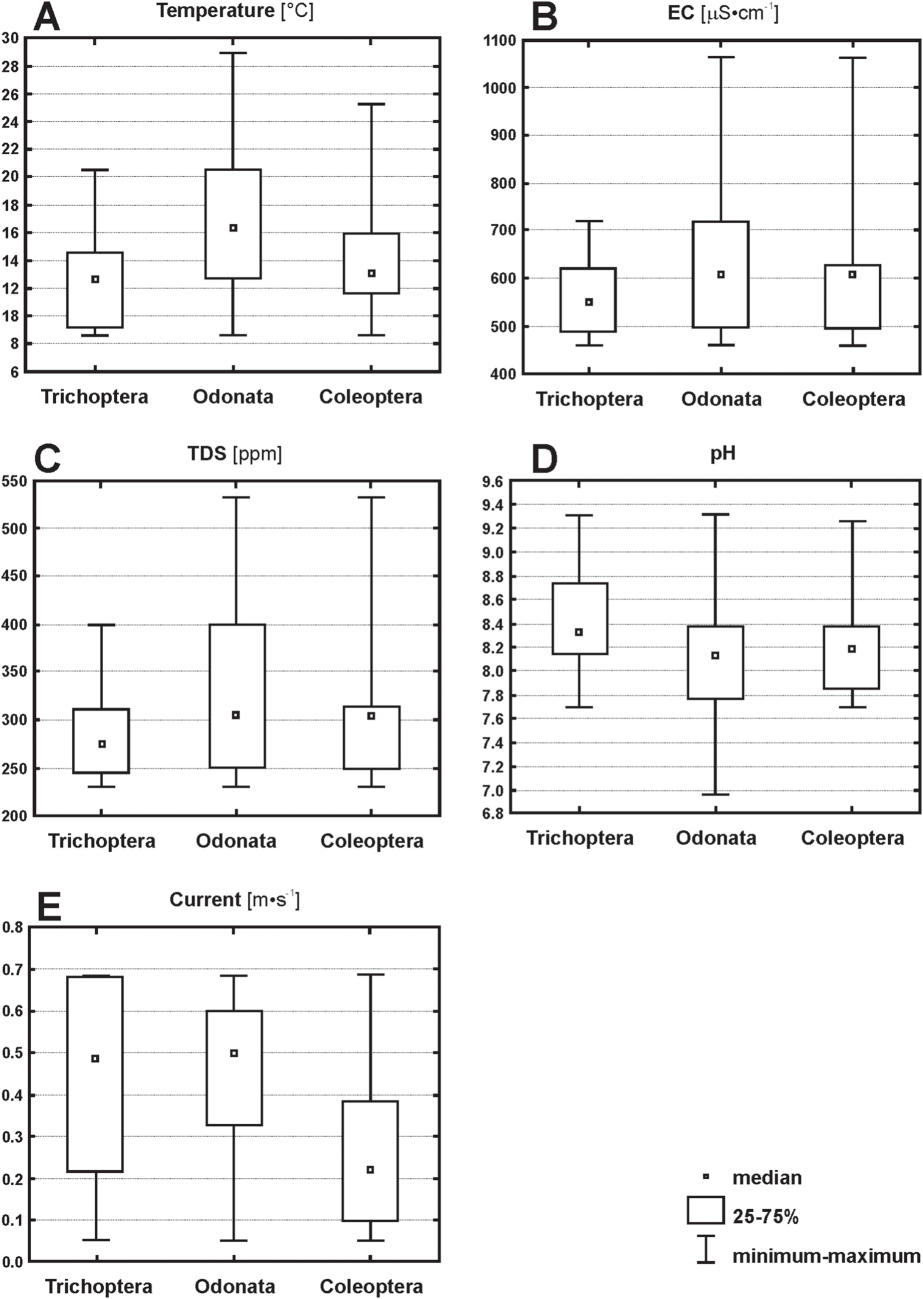
Changes of abundances of Trichoptera, Odonata and Coleoptera along five environmental gradients (with Kruskal–Wallis test). (A) Temperature (*H* = 21, *p* = 0.00001). (B) EC (*H* = 8.3, *p* = 0.01). (C) TDS (*H* = 8.6, *p* = 0.001). (D) pH (*H* = 7, *p* = 0.001) (E) Current (*H* = 18, *p* = 0.001).

A comparison of the mean abundances of particular groups with five significant, separate water parameters revealed certain regularities within their ranges (Fig. 8). All the differences found among the OCT fauna were statistically significant. Dragonflies occurred within the widest temperature spectrum—from 8.6 °C to 29 °C, while the spectrum for caddisflies within the narrowest—from 8.6 °C to 20.5 °C and in the intermediate spectrum—from 8.6 °C to 25 °C; the mean temperatures for three orders were thus 16, 12 and 13 °C, respectively. In the case of EC, dragonflies and beetles exhibited an identical occurrence pattern: from 461 to 1,063 μS/cm (mean 601 μS/cm), whereby dragonflies were the most numerous in the 500–700 μS/cm interval, and beetles in the 500–600 μS/cm interval; caddisflies, by contrast, did not tolerate values higher than 720 μS/cm. Very similar relationships were found for TDS: dragonflies and beetles occurred to an upper limit of 532 ppm, caddisflies to 400 ppm. All three orders were present within the same range of current speeds. Caddisflies were distributed more evenly in habitats with both a faster and a slower current, whereas beetles exhibited a positive association with slower flowing waters. In the case of pH, dragonflies were found at the lowest recorded values (6.9), while caddisflies and beetles were only present at pH > 7.7. In general, caddisflies were associated with a slightly higher pH than the other two orders.

## Discussion

### Environmental factors influencing entomofauna of industrial canal system

The influence on aquatic insects of elevated surface water temperature, caused by both natural and anthropogenic factors, has long been of interest to researchers, as it may disrupt their metabolic and growth rates, feeding, reproductive capacity, life cycles and distribution (Stewart et al., 2013; Dallas & Ross-Gillespie, 2015). Presently, in the face of intensifying human pressure and altered thermal regimes in waters due to climate warming (Haidekker & Hering, 2008), it is particularly important that reference conditions be effectively determined for a given habitat type or group of organisms reacting to individual stressors or a combination of them. Although the concept of bioindicators for the aquatic environment was introduced more than 100 years ago, their definition and the question of harmful factors need to be reevaluated, something that Sahlén & Ekestubbe (2001) drew attention to in their work on dragonflies.

The results of the present study indicate that the factor differentiating the occurrence of insects in the outlet canals was temperature—as was anticipated. In 2013 water temperatures were as high as 30 °C, which was likely to eliminate all caddisflies and nearly all beetles from it due to their thermal intolerance. Dragonflies reacted in the opposite way: the number of species and individuals rose markedly, however, in contrast to the fauna of the inlet canals, these were exclusively eurytopic species, including thermophilous ones (*Erythromma viridulum*, *Orthetrum albistylum*) (Bernard, Buczyński & Tończyk, 2002). To a large extent these results corresponded with the literature: Stewart et al. (2013) state that the mean upper thermal tolerance (UTT) for caddisflies is 31 °C while for dragonflies it is 41.9 °C. In the light of those results, it is of particular interest that beetles, though they are even more tolerant to heat (UTT = 43.4 °C), were very few in number and represented by just two species. Moreover, whereas stenotopic species (mostly rheophiles) were dominating in the river and inlet canals, it was almost exclusively eurytopic species that occurred in the outlet canal (Klausnitzer, 1996), mostly typical of the warm, shallow shores of standing waters. One cannot rule out the possibility that the elevated temperature of the cooling waters, besides its direct effect on insects (lethal to caddisflies), could have intensified the effect of some toxic compound present in the water (Cairns, Heath & Parker, 1975), which was lethal to beetles.

The key factor underlying OCT distribution in the river was EC, while in the river-fed inlet canals there were two such factors—EC and salinity. These results demonstrate that, despite the differences in the habitats (natural vs. artificial, non-impacted vs. impacted), they support a roughly similar fauna governed by the same or similar drivers. This medium-level qualitative faunistic similarity is depicted by the cladogram (Fig. 4). In this case the physical and chemical properties of the water and hydrological continuity were the most important for the fauna. However, examination of these similarities at more detailed NMDS and also quantitative SIMPER levels shows each of the three main site types to be quite different: there are thus significant links between given types of habitat, but with each retaining a specific distinctiveness.

The insect assemblage colonizing the outlet canals was governed by oxygen and, especially, by a structural factor: aquatic vegetation. Since temperature was the key factor for the insects, and oxygen is very highly correlated with it, as the CCA plot indicates this system is influenced by overlapping factors, described in studies on disturbed ecosystems by Fernández-Aláez et al. (2002) and Kefford (1998). This often leads to such masked factors being overlooked or underestimated. In general, the removal of such highly correlated but different parameters prior to the analyses proper requires great caution. If we decide to do this we must remember that the response of invertebrates refers indeed to two factors and the reference to only one of them can be misleading. The distribution of dragonflies—the dominant in the outlet canals owing to their tolerance to physical and chemical factors—also depends on the structure and heterogeneity of the habitat. It is worth noting that the degree of the bottom macrophyte overgrowth as well as the current were the most significant factors influencing the distribution of a river’s dragonflies after dredging (Buczyński et al., 2016). Here, too, *Ischnura elegans* was the most intimately associated with the aquatic vegetation. The example of these outlet canals shows that, even in the most disturbed artificial habitats, species are sensitive to multiple habitat components (i.e., water properties, hydromorphology and vegetation of the watercourse), which should be taken into account and not be neglected during the monitoring of such habitats. Also, this is an indication of the considerable adaptive capabilities of dragonflies in relation to the cyclic disturbances that their populations may be exposed to. Abdul et al. (2017) have drawn attention to this aspect in studies of dragonfly assemblages of a polluted river: dragonflies developed many morphological and physiological adaptations in order to counter the deterioration in habitat quality.

At the level of the particular insect groups, dragonflies and caddisflies were dependent on the same physical and chemical factors in the river: EC and salinity, while beetles only on EC. In the inlet and outlet canals only dragonflies showed significant response: their assemblages were affected by oxygen and current. This shows that while these orders do have many general traits or reactions in common (Haidekker & Hering, 2008; Stewart et al., 2013; Chang et al., 2014), they can be influenced by different parameters at different types of habitats. This provides sufficient justification for performing studies covering not one but many different taxonomic groupings, as analysis of their reactions to environmental changes yields more comprehensive ecological information.

### The response of aquatic insects to dredging impact

The effect of dredging is best followed in the inlet canals. Two years after impact, total diversity, abundances and species richness of dragonflies and beetles were higher than before impact, and the number of beetle species increased 2.5 times. Interestingly, the data from the inlet canals coincide exactly with the reactions of an assemblage of dragonflies and beetles to dredging in a small, regulated, lowland river (Buczyński et al., 2016; Dąbkowski et al., 2016). A similar result had been expected for the outlet canal, but was the exact opposite.

In the case of caddisflies, the number of individuals and species in 2013 was lower than before dredging, which may testify to the weaker recolonization potential of this group and its stricter habitat requirements. In a study of the effect of dredging in the artificial habitat of the navigation channel of the Columbia River, McCabe et al. (1998) also found that total taxa richness after dredging increased, but that neither the mean diversity index (H) nor the evenness (E) of the benthic assemblages changed in a statistically significant manner, which corresponds with the present results. The results of BACI interactions showed that all faunistic metrics after impact remained at the same level. Similar results relating to the Shannon–Wiener diversity index had been obtained for dragonflies (Buczyński et al., 2016) and caddisflies (Zawal et al., 2016) in a small dredged river. In the present case, only the ANOVA results of fauna in the canals revealed significant differences in mean species richness and dominance before and after dredging. Albeit this result was governed mostly by the site effect. Faunistic metrics as a whole do not always enable a complex mixture of impacts to be evaluated (Fernández-Aláez et al., 2002). Relatedly, McCabe et al. (1998) found that after dredging, benthic invertebrates were able to colonize the area quite rapidly and that, generally speaking, the impact did not have much of a negative effect on the canal’s fauna; this could have been due to the rapid rate of recolonization and the small scale of the dredging.

The time required by fauna to recover after impact can vary widely—from a few weeks to a few years—and depends on numerous factors and on the assemblage affected (Wallace, 1990; Yount & Niemi, 1990). In the present case, the canals were studied 2 years after dredging had commenced, a process that almost completely eradicated the previous ecosystem. The insects had to colonize the new habitat via drifting and through the air. The different accessibility of these pathways of colonization to the various habitats is likely to have affected its success. Site 2 was in constant hydrological contact with the Kurówka River, thus there was likely less restrictions on drifting. To reach site 3, the water had to pass through a pumping station which was a significant obstacle for most insect larvae. Sites 4 and 5 could be colonized solely from the air. If drifting was possible, then the level of colonization was high, but this level was much lower at the sites where drifting from the river was limited or non-existent, with only the strongest fliers, that is, dragonflies, achieving a measure of success (Corbet, 1999); beetles, amongst which there are both strong and weak fliers, were less successful (Buczyński & Przewoźny, 2010). Caddisflies were the least successful.

Since the factors shaping an invertebrate assemblage are not only abiotic but include its surrounding environment (Suriano et al., 2011), insects migrated from the whole river valley and its waters to where the canals were situated. Dragonflies, the strongest fliers that are present over a very wide spectrum of physical and chemical water properties, occurred in both types of canal. The inlet canal (site 2) was dominated by beetles, while non-case-making caddisflies were replaced by members of the *Mystacides* genus. Unfortunately, given the generally small number of caddisflies at this site, trend information is unavailable, however a response such as this to dredging was not found in other studies of the impact of this process on trichopterans. Indeed, the reverse in some cases—the abundance and species richness of non-case-making genera (*Hydropsyche*, *Neureclipsis*) increased abruptly after impact (Zawal et al., 2016). Moreover, the beetle and caddisfly assemblage of the other inlet canal (site 3) had not yet reconstituted itself by 2013; in the intervening two years the structure of the aquatic vegetation with islands of detritus had not yet been restored. In this situation, species entirely dependent on these factors regarding food or habitat, for example, *Limnephilus lunatus* or *Halesus* spp. (Graf et al., 2008), could not return. Zawal et al. (2016) also pointed out the appreciable loss of caddisfly species associated with vegetation after dredging. On the other hand, hitherto unrecorded rheophils appeared in the inlet canals (Bernard et al., 2009): *Gomphus vulgatissimus* (sites 2 and 3) and *Ophiogomphus cecilia* (site 3), which made up quite a large proportion of the material acquired from site 3. This may have been due to the removal during dredging of the muddy sediments, unsuitable for these species, which prefer a mineral bottom with a species-dependent grain size and a much smaller proportion of organic matter than on a muddy bottom (Müller, 1995). A similar effect of dredging just a short time after impact was reported by Buczyński et al. (2016).

A typical effect of dredging is the replacement of some species with others (Zawal et al., 2016; Buczyński et al., 2016): in the canal system studied here, there was a particularly conspicuous species shift of dragonflies in the cooling waters. After impact, *Ischnura elegans* appeared in large numbers on the outlet canals, accompanied by smaller numbers of *Erythromma najas*, *E. viridulum* and *Orthetrum albistylum. Orthetrum albistylum* prefers vegetation-poor waters, with exposed banks and a high temperature (Bernard et al., 2009), so the removal of vegetation from the outlet canals during dredging clearly created optimal conditions for this species. In 2013, aquatic and shore vegetation were reestablished, which in turn was favourable to phytophilic Zygoptera: *Ischnura elegans* and *Erythromma* spp. These species are widespread in the river valleys of eastern Poland, especially in oxbow lakes, a great many of which lie close to the study area (e.g., Buczyński, 2006; Buczyńska, Buczyński & Lechowski, 2007). Thus, their colonization of the dredged canal once plant-life had started to return was expected. *Ischnura elegans*, dominant after dredging, is capable of dynamic and long-distance dispersal (Parr, 1973). But dispersal abilities, especially given the small distances separating the canals from natural waters, do not explain the observed differences. Among the *Erythromma* species, recorded in small numbers, *E. viridulum* is also expansive, but *E. najas* is not regarded as such (Hassall, Thompson & Harvey, 2009; Watts, Keat & Thompson, 2010). The colonization success of *Ischnura elegans* should be seen in the light of its less restrictive habitat requirements, which is why it is generally a more common species and may thus arrive from a larger number of potential donor habitats, and consequently it lacks a microhabitat specialization. *Ischnura elegans* can develop in vegetation of a very varied structure, whereas *Erythromma* spp. are associated with nympheids or submerged vegetation, periodically appearing on the surface (e.g., *Myriophyllum* spp., *Ceratophyllum* spp.) (Bernard et al., 2009), but of which there is little in the early stages of succession.

### The tolerance patterns of Odonata, Coleoptera and Trichoptera in an industrial water system

The three orders reacted to particular parameters differently: trichopterans were the most sensitive in this particular ecosystem, with high temperatures likely inhibiting recolonization or causing high mortality. High ECs could have also been harmful to some species, although many species are known to tolerate values far greater than 700 μS/cm (Basaguren & Orive, 1990; Gerecke, 1991; Gallardo-Mayenco, 1994), which was the upper limit in the canals studied here. Dragonflies were present in the broadest ranges of physical and chemical factors. Beetles, a group situated between dragonflies and caddisflies, demonstrated responses resembling those of dragonflies (to EC, TDS), others were closer to caddisflies (temperature, pH). Beetles are exceptionally resistant to high values of salinity and EC, as a study of highly saline periodic waters in Sicily demonstrated (Gerecke, 1991). This resistance applies primarily to imagines, which, should conditions become intolerable, can migrate to other habitats. According to Chang et al. (2014), Trichoptera had significantly lower pollution tolerance values than Odonata which, in turn, were the less tolerant group than Coleoptera. Our findings confirm that caddisflies are the most sensitive, however, dragonflies had broader tolerance ranges of temperature, EC and TDS than beetles. Still, mean tolerance values of EC and TDS as well as their upper and lower limits were identical for Odonata and Coleoptera.

The results of this study give interesting insight into how one analyses complex hydrological systems in which various stressors act and overlap. In such conditions, each order of organisms contributes significantly to the general response. These results have demonstrated that caddisflies are unsuitable for detecting changes in constant temperatures above 20 °C, which corresponds to the discovery of Stewart et al. (2013), that the thermal tolerance of the fauna suggests 21 °C as the UTT for a range of sensitive freshwater insect taxa. On the other hand, they were found to be the best order for differentiating natural and artificial habitats (here—the control site vs. inlet canals). Dragonflies, by contrast, are best suited for tracking faunal changes in the canals themselves, being sensitive to both water parameters and the structural aspects of habitats; in addition, they are thermally the most resistant, which paradoxically might favor certain species in the cooling waters. According to Hassall & Thompson (2008) odonate assemblages exhibit high rates of species turnover in response to increasing temperatures, moreover, they are good indicators of certain water pollutants whose concentrations increase in higher temperatures. Thus, the monitoring of dragonfly populations in industrially heated waters would bring important information about the ecological status of such ecosystems and individual species could be considered good thermoindicators. The tolerance ranges of beetles are narrower than those of dragonflies, and some parameters may well be limiting in their case. With respect to general indices (species richness or abundance), beetles do not show differences between natural and artificial waters, however, they were the most numerous organisms recolonizing the inlet canals after they had been dredged.

### Investigating and monitoring of industrial waters using entomofauna—practical issues

This study has shown that artificial, industrial watercourses are interesting research objects, the fauna of which is shaped not only by the physical and chemical parameters of the water, but also by structural factors in the watercourses themselves. The OTC assemblages of the canals are linked with the fauna of the river hydrologically feeding the whole industrial system, however these linkages did not influence all species occupations. In our case, human-impacted alteration were beneficial for some species, especially dragonflies. In general, the potential bioindication value of the studied insect groups is varied. Basing on our study, it appears that managers may face counter-intuitive or diametrically opposed actions based on the species of interest. The selection of the appropriate taxonomic group preceded by the recognition of habitat conditions (water analysis, hydromorphological factors) is crucial for the biological monitoring of specific sites or habitats. For example, dragonflies are the best option for canals carrying heated water. In waters with an EC higher than 720 μS/cm, dragonflies and beetles can be used. The assessment of the ecological status of the ecosystem after dredging can be tracked using all insect groups. Even in habitats so heavily impacted by intentional modifying of structural elements (e.g., aquatic vegetation) one can contribute to increasing the heterogeneity of watercourses and the number of niches available for species with different requirements. In this way, we contribute to the protection of the local biodiversity, and canals—despite their original industrial purpose—can be an important element of the habitat network for hydrobionts (Buczyński, 2015).

The dredging process itself affected all three orders. The reaction of dragonflies and beetles was similar, resembling that observed in a small river (Buczyński et al., 2016; Dąbkowski et al., 2016). Interestingly, caddisflies reacted in the opposite manner, likely due to their reduced dispersal potential, the destruction of significant aspects of their habitat, or simply the longer period of time they require to rebuild their assemblage. If any one of these insect orders were to be excluded from the analysis, we would miss important data. Likewise, relying solely on faunistic metrics does not yield the full impacts of dredging either: for instance, the diversity or dominance index may be misleading, as it does not take into consideration the crucial aspect of species shift after impact (here, dredging), which the SIMPER analysis revealed. That is why, when investigating the dependences in complex systems like the present one, utilizing the species level is a must. It is extremely important in hydrobiology that a set of metrics be developed that takes functional traits relating to a more refined taxonomic level into account (Suriano et al., 2011).

When studying habitats subject to very high levels of industrial anthropic pressure, it is well to bear in mind possible limitations inherent in the type of impact or disturbance.

## Conclusions

Our research highlighted that running waters of anthropogenic origin used in industry, influenced by strong and regular transformations (heated waters, dredging) make up an interesting model study system. Such systems may be important components in the hydrological network at local and regional scales, as they themselves are subject to local and regional processes of species occupation and assembly. Indeed, our study has shown that these locations have specific assemblages of aquatic entomofauna, dependent not only on physical and chemical properties of water (EC, salinity, DO, current) but also on the structural factors of the canals (the coverage of aquatic vegetation).

Total species richness of OTC increased after dredging in the canals and statistically significant species shift was observed. In turn, changes in time and space reflected in faunistic metrics (BACI interactions) were significant only in the case of mean species richness and the dominance index between inlet and outlet canals.

Tolerance ranges of particular insect orders differed on five key physical and chemical water parameters differed: caddisflies were the most sensitive, then beetles and dragonflies. These suggest that the comprehensive response of entomofauna to environmental transformations and/or gradients should not be limited to a single taxonomic group or a level of insect organization because such results can be inaccurate. In this light our studies are not only fundamental but also applicable, which is particularly important in aquatic man-made habitats that are often neglected by researchers.

Even in habitats as heavily impacted as industrial canals, environmental disturbances do not have to be negative only. For example: dredging increased the total species richness of insects and heated waters were favorable for some thermophilous dragonflies. It is worth emphasizing that in this type of habitat the effects of negative factors affecting the entomofauna may overlap, therefore each case should be analyzed with caution. Consequences of these changes on the ecosystem should be addressed by further research, involving other taxonomic groups and stressors.

## Supplemental Information

10.7717/peerj.6215/supp-1 Supplemental Information 1 Database: species and environmental variables. A. Sample. B. Subsample. C. Species. D. Species code. E. Insect order. F. Site. G. Year. H. Month. I. Number of species. J. Temperature. K. pH. L. Dissolved oxygen. M. Electrolytic conductivity. N. Total dissolved solids. O. Salinity. P. Current. Q. Aquatic plants. R. Riparian plants.

## Additional Information and Declarations

### Field Study Permissions

The following information was supplied relating to field study approvals (i.e., approving body and any reference numbers): Field permit was approved by: Grupa Azoty Zakłady Azotowe “Puławy” S.A. (project number ZK/6011/255/2018). The Regional Directorate for the Environmental Protection in Lublin confirmed that studies covering running waters which are not legally protected or insect species which are not legally protected in Poland, do not require the permission.

### Data Availability

The following information was supplied regarding data availability: The raw data is available in the Supplemental File.
